# Gender Differences in the Physical and Psychological Manifestation of Childhood Trauma and/or Adversity in People with Psychosis

**DOI:** 10.3389/fpsyg.2015.01768

**Published:** 2015-11-23

**Authors:** Shaun Sweeney, Tracy Air, Lana Zannettino, Sonal S. Shah, Cherrie Galletly

**Affiliations:** ^1^Discipline of Psychiatry, University of Adelaide, AdelaideSA, Australia; ^2^School of Nursing and Midwifery, Flinders University, AdelaideSA, Australia; ^3^Neuropsychiatric Epidemiology Research Unit, School of Psychiatry and Clinical Neurosciences Laboratory, University of Western AustraliaPerth, WA, Australia

**Keywords:** childhood trauma and/or adversity, psychosis, self-reported health status, gender

## Abstract

The link between childhood trauma and/or adversity and risk of psychosis is well known. Our aim was to determine the prevalence of childhood trauma and/or adversity in people who have psychotic disorders and to investigate the association between childhood trauma and/or adversity and a range of social and health measures. Participants (*n* = 391, 42% male) were specifically asked about any experience of childhood trauma and/or adversity. Respondents provided information about education, employment, physical health, and health service utilization. Univariate analyses revealed that childhood trauma and/or adversity was associated with poorer levels of self-reported physical health and social problems. This includes the experience of chronic pain, headaches, arthritis, asthma, and victimization/stigma in men. Participants with a childhood trauma and/or adversity history indicated higher rates of lifetime suicide attempts with women reporting more lifetime depressive symptoms. Multivariate analyses revealed differing profiles in relation to physical and psychological health variable between males and females. Males with the experience of childhood trauma and/or adversity were significantly more likely to report cardiovascular/stroke issues, migraines and anhedonia. Females with the experience of childhood trauma and/or adversity were more likely to report a lifetime history of elevated mood and to be married or in a *de facto* relationship. There has been very little research into the assessment and treatment of the effects of childhood trauma and/or adversity in adults with psychosis. Childhood trauma and/or adversity may contribute to higher rates of self-reported poor health in men and is associated with increased depression in women. Our findings suggest that interventions to address the effects of past trauma are urgently needed.

## Introduction

Psychosis is defined as the presence of hallucinations and delusions and is present in a number of psychiatric disorders including schizophrenia, delusional disorders, schizoaffective disorders, and drug-induced psychosis. These symptoms can result in impaired functioning in work and school, and can affect parenting, self-care, independent living, interpersonal relationships, and leisure time (Mueser and McGurk, 2004). Psychotic disorders are complex illnesses that can have a profound long-term health, psychological and social impact on the individual. Research in the field indicates that complex, interrelated factors can determine the etiology and course of the illness. There is also strong evidence pointing to the relationship between the experiences of trauma and/or adversity and the development of psychosis, including a correlation between trauma and/or adversity in childhood and the development of psychosis (Morgan and Fisher, 2007).

Estimates of the rates of childhood trauma and/or adversity (CTA) worldwide indicate that every year millions of children experience trauma including physical, sexual, emotional abuse, and severe neglect (Krause, 2008). The terminology associated with trauma, adversity, and early life stress is often used interchangeably across numerous social and health contexts. In discussing the different interpretations of trauma and adversity, Brown et al. (1999) provide a definitive definition of trauma and adversity in childhood that encompasses child abuse and neglect. This definition includes the experience of verbal assaults on a child’s sense of worth, bodily assaults that pose risk of injury, sexual contact, failure to provide basic psychological/emotional needs, and a failure to provide basic needs (Bernstein and Fink, 1998; Browne and Winkelman, 2007). CTA is associated with a range of poor health and psychological outcomes, (Spataro et al., 2004) and is associated with the development of psychotic disorders in adulthood (Janssen et al., 2004; Matheson et al., 2013). In particular, the experience(s) of CTA can lead to high levels of anxiety and is associated with hallucinations, psychological withdrawal, depression, and hopelessness (Lysaker and Salyers, 2007). The health and psychosocial outcomes of CTA can vary according to gender, with women being more likely to experience sexual abuse whilst men tend to be subjected to physical abuse and/or bullying (Read et al., 2005). Additionally, in particular cohorts, such as adult women in drug treatment services, as many as 62–81% report CTA (Teets, 1995; Gil-Rivas et al., 1997; Liebschutz et al., 2002).

While research has been conducted into the influence of CTA, particularly child sexual abuse on health, psychological, and social functioning in female populations, CTA in male cohorts has received less attention, primarily due to its seemingly lower prevalence rates and less overt symptomatic presentation(s). There is widespread evidence that psychosis is correlated with poor physical health, lower levels of educational attainment, and poorer socioeconomic outcomes (Lund et al., 2010). People with psychosis can also exhibit higher rates of obesity (Taylor et al., 2012), illness comorbidity (Sim et al., 2004), and substance abuse than in the general population (Addington and Addington, 2007). For those with a history of CTA and who have developed a psychotic disorder, the combination of this serious mental illness along with the challenges of living with the psychological effects of CTA can make health, economic and social functioning extremely difficult (Spataro et al., 2004).

Despite an extensive collection of research into the effects of psychosis on many of the domains of life there has been little exploration of the physical impact of CTA on people living with psychosis. The present study took place in the northern region of Adelaide, South Australia. This is a disadvantaged region with a high proportion of people in receipt of government benefits, high unemployment rates, lower levels of education, and high rates of mental health (Ahern et al., 2006). Within this adverse socioeconomic environment, our aim was to determine the prevalence of CTA in people who have psychotic disorders, and to investigate the associations between CTA and a range of psychiatric, social, and health measures. Given that gender differences in both the nature and adult consequences of CTA were likely, we undertook separate analyses for men and for women.

It was expected that co-existing CTA and psychosis would be associated with a poorer outcome than psychosis alone. Additionally, the role of gender in affecting psychosocial and health outcomes was also explored. We expected participants who experienced CTA to have left school earlier, be more likely to be unemployed, and to have poorer economic functioning. We also expected people subject to CTA to be more likely to abuse drugs and alcohol, self-harm and/or attempt suicide. Given their exposure to abuse and/or violence during childhood, we also considered it likely that they would experience stigma, bullying, and/or violence as adults. We also expected that their physical health (both objective and subjective) would be worse. These assumptions are based on evidence in the CTA literature indicating the correlation between CTA and lower socio-economic status, and poor health compared to non-CTA populations.

## Materials and Methods

Participants for the study were randomly selected from people who have been in contact with a mental health service or a non-government organization funded to provide mental health services. The SHIP projected utilized a psychosis screener to identify potential participants. Potential participants were screened for psychosis between April 1st, 2009 and March 31st, 2010. The psychosis screener identified potential SHIP participants who were positive for psychosis on the basis of their contact(s) with mental health services or who recorded ICD-10 diagnosis of psychosis. The psychosis screener identified 1825 adults aged 18–64 years who were residents in the South Australian postcode catchment area. Potential participants needed to have been in contact with public mental health services in the 12 months prior to the survey to be eligible to participate in the SHIP study. The exclusion criteria included severe cognitive impairment, the inability to comprehend English sufficiently to complete the interview without the use of an interpreter. Attempts were made to recruit all of these potential participants. Eight hundred and twenty three were unable to be contacted due to change of personal circumstance (e.g., moved away from the catchment area, changed their phone number, were non-responders to phone/mail contact or did not attend the SHIP interview), 16 were known to have died, 33 did not meet inclusion criteria due to inability to sufficiently communicate in English, 42 did not have capacity to give informed consent, and 507 refused. Potential participants were identified during the SHIP census period and in total 402 participants were interviewed. Comparison of screening data for interviewed participants and those selected for interview but not participating for any reason indicated no systematic selection biases. Both groups were alike in terms of sex (60% of those interviewed were male compared to 62% of those selected but not interviewed) and age group (44% of those interviewed were aged 18–34 years at the time of screening compared with 43% of those not interviewed). The psychosis screening profiles for both groups were similar indicating no marked differences in terms of lifetime symptom profiles based on the screener items.

The SHIP interview schedule consisted of 32 modules and asked participants about psychopathology, substance use, physical health, functioning, disability and quality of life, education, employment, accommodation, and childhood adversity. The social, health, and economic profile information of participants consisted of, but were not limited to: demographic status, socioeconomic and psychosocial status, health and physical functioning, diagnosis, and symptomology. Diagnoses were made using the Diagnostic Interview for Psychosis (DIP; Castle et al., 2006). The DIP contains selected interview questions and probes from the WHO Schedules for Clinical Assessment in Neuropsychiatry (Wing et al., 1990) mapped onto the 90 diagnostic items of the operational criteria checklist for psychotic and affective illness (McGuffin et al., 1991). The DIP measured both lifetime and current illness symptoms for psychosis. A computer algorithm provided the diagnostic classification in accordance with ICD-10, DSM-IV, and other criteria on the basis of the DIP scores. This reduced the subjective bias in the interpretation of symptoms and signs (Shah et al., 2014). No specific analysis was conducted regarding the non-responders to the SHIP research project. Further information regarding the method of the SHIP research project is detailed in Morgan et al. (2012).

Questions regarding the occurrence and nature of CTA were included in the interview. In Australia, all states follow a national framework of guidelines for defining the four types of child abuse (a) sexual abuse, (b) physical abuse, (c) emotional abuse, and (d) neglect. The classification of reported CTA was undertaken in collaboration with staff from the Western Australian Department of Child Protection to ensure conformity to national guidelines. This paper includes only those cases where the CTA is consistent with the standard Australian definitions, took place when the participant was aged 18 years or younger and occurred prior to the onset of psychotic illness.

### Ethics Statement

Ethics approval for this research was obtained from the Human Research Ethics Committee of the Queen Elizabeth Hospital (Protocol number: 2009179).

### Assessments

The Second Australian Survey of Psychosis 2010 interview schedule was comprised of 32 modules, including the Alcohol Use Disorders Identification Test (Babor et al., 2001), the World Health Organization Schedules for Clinical Assessment in Neuropsychiatry (Kirkpatrick et al., 1989; World Health Organization, 1999). The details for all other measures are described in Morgan et al. (2012).

### Childhood Trauma and/or Adversity

Questions about the occurrence and nature of CTA were included in the SHIP interview. CTA was coded on the basis of responses to the question: “Were there any other very distressing or traumatic events in your childhood (not including parental separation or divorce, or loss of a close relative)?”

### Statistical Analyses

Statistical analyses were performed using Stata, version 12 (StataCorp, 2011). A two-step model building procedure was used to determine variables associated with CTA. In the first step, we used univariate analyses (chi-squares and *t*-tests) to examine relationships between CTA and independent variables. In the second step, we used multivariate logistic regressions, including only variables that were significantly associated with CTA status at alpha ≤0.05 in the first step. As the incidence of CTA was much higher in females, the analyses were also stratified by gender to explore gender specific associations. In the final stratified model, only the significant variables were retained, adjusting for possible confounders of health status such as lifetime histories of smoking, heavy alcohol usage and cannabis use.

Given the difference between genders in the numbers reporting CTA, a *post hoc* power analysis with the program G^∗^Power, Version 3.1.9.2 (Faul et al., 2009) was conducted. The analysis implied that statistical power (power = 0.96) was sufficient, rendering it unlikely that significant differences between the groups could have been overlooked.

## Results

Of the 391 participants, 59.3% of reported experiencing CTA. CTA was reported more in females than males (χ^2^ = 19.7, *df* = 1, *p* < 0.001). **Table 1** depicts the results of the univariate analyses comparing CTA and no CTA participants on socio-demographic data. Participants who had experienced CTA were more likely to be married or living in a *de facto* relationship (χ^2^ = 13.2, *df* = 2, *p* = 0.001), and had left school earlier (*t* = –3.22, *df* = 389, *p* = 0.001). There was no difference in diagnosis or in rates of current smoking, lifetime history of heavy alcohol use, or cannabis use, between those with and without CTA.

**Table 1:** Socio-demographic data for people with psychosis, comparing those who had experienced childhood trauma and/or adversity (CTA) with those who had not.

	CTA (*n* = 232)		No CTA (*n* = 159)		Total (*n* = 391)		*p*
**Age and sex**							
Female (*n*, %)	118	50.9	45	28.3	163	41.7	<0.001
Age (mean ± SD, years)	39.1	10.2	37.5	11.1	38.4	10.5	0.16
Age left school (mean ± SD, years)	15.8	1.3	16.3	1.2	16.0	1.3	0.001
**Marital status (*n*, %)**							
Single	106	45.7	102	84.6	208	53.2	0.001
Married/*de facto*	59	25.4	24	15.1	83	21.2	
Currently separated, divorced, widowed	67	28.9	33	20.8	100	25.5	
**Income (*n*, %)**							
Main income source: pension	212	91.8	147	93.0	359	92.3	0.65
Duration of illness (years)	15.3	10.0	13.2	9.4	14.4	9.8	0.043
**Employment (*n*, %)**							
Paid Employment (past year)	54	23.3	38	23.9	92	23.5	0.89
Voluntary/Unpaid work (past 12 months)	0.34	14.7	20	12.6	54	13.8	0.56

### Physical and Psychological Health

**Table 2** displays the results for the univariate analyses on physical health. The CTA group generally indicated higher rates of reported lifetime physical health conditions. More of the CTA positive group experienced chronic pain (χ^2^ = 18.24, *df* = 1, *p* = 0.001), headaches/migraines (χ^2^ = 19.73, *df* = 1, *p* = 0.001), arthritis (χ^2^ = 10.88, *df* = 1, *p* = 0.001) and asthma (χ^2^ = 5.52, *df* = 1, *p* = 0.019). In the overall sample, cardiovascular disease and/or stroke was also more common in the CTA positive group (χ^2^ = 9.80, *df* = 1, *p* = 0.002).

**Table 2:** Lifetime physical conditions.

	CTA (*n* = 232)		No CTA (*n* = 159)		Total (*n* = 391)		*p*
**Lifetime physical conditions (*n*, %)**							
Chronic pain	101	43.5	35	22.4	136	35.1	<0.001
Asthma	81	34.9	37	23.7	118	30.4	0.019
Cardiovascular/stroke	54	23.5	17	10.9	71	18.4	0.002
Severe headaches/migraines	89	38.4	27	17.3	116	29.9	<0.001
Arthritis	64	27.6	21	13.5	85	21.9	0.001
Diabetes	56	24.5	39	25.2	95	24.7	0.88
Epilepsy	25	10.8	10	6.5	35	9.0	0.15
Cancer	11	4.7	4	2.6	15	3.9	0.28
**Males (*n*, %)**							
Chronic pain	41	36.0	25	22.1	66	29.1	0.022
Asthma	33	29.0	24	21.2	57	25.1	0.18
Cardiovascular/stroke	27	23.7	11	9.7	38	16.7	0.005
Severe headaches/migraines	39	34.2	18	15.9	57	25.1	0.001
Arthritis	20	17.5	9	8.0	29	12.8	0.031
Diabetes	26	23.2	24	21.4	50	22.3	0.75
Epilepsy	18	15.8	7	6.2	25	11.0	0.021
Cancer	2	1.8	3	2.7	5	2.2	0.64
**Females (*n*, %)**							
Chronic pain	60	50.9	10	23.3	70	43.5	0.002
Asthma	48	40.7	13	30.2	61	37.9	0.23
Cardiovascular/stroke	27	23.3	6	14.0	33	20.8	0.20
Severe headaches/migraines	50	42.4	9	20.9	59	36.7	0.012
Arthritis	44	37.3	12	27.9	56	34.8	0.27
Diabetes	30	25.6	15	34.9	45	28.1	0.25
Epilepsy	7	5.9	3	7.1	10	6.3	0.78
Cancer	9	7.6	1	2.3	10	6.2	0.21

More males with a history of CTA than expected reported chronic pain (χ^2^ = 5.2, *df* = 1, *p* = 0.022), cardiovascular disease/stroke (χ^2^ = 7.9, *df* = 1, *p* = 0.005), headaches/migraines (χ^2^ = 10.0, *df* = 1, *p* = 0.001), arthritis (χ^2^ = 4.7, *df* = 1, *p* = 0.031) and epilepsy (χ^2^ = 5.3, *df* = 1, *p* = 0.021). More females with CTA than expected reported chronic pain (χ^2^ = 9.8, *df* = 1, *p* = 0.002) and headaches/migraines (χ^2^ = 6.2, *df* = 1, *p* = 0.012).

Univariate analyses revealed that the CTA positive group experienced different rates of lifetime psychological symptoms than the non-CTA group (**Table 3**). For example, the CTA group were more likely to report a lifetime history of depressive mood (χ^2^ = 7.96, *df* = 1, *p* = 0.005), anhedonia (χ^2^ = 14.30, *df* = 1, *p* = 0.001), poor concentration (χ^2^ = 4.67, *df* = 1, *p* = 0.031), elevated mood (χ^2^ = 5.16, *df* = 1, *p* = 0.023). However, the non-CTA group were more likely to report a lack of insight (χ^2^ = 5.68, *df* = 1, *p* = 0.017). Males with CTA also were more likely to have experienced anhedonia (χ^2^ = 8.44, *df* = 1, *p* = 0.004) and subjective thought disorder (χ^2^ = 3.87, *df* = 1, *p* = 0.049). Conversely, females with CTA were more likely to have reported depressive mood symptoms (χ^2^ = 5.59, *df* = 1, *p* = 0.018) and elevated mood symptoms (χ^2^ = 4.30, *df* = 1, *p* = 0.038).

**Table 3:** Psychological symptoms: lifetime.

	CTA (*n* = 232)		No CTA (*n* = 159)		Total (*n* = 391)		*p*
**Lifetime symptoms (*n*, %)**						
Delusions	213	91.8	148	93.1	361	92.3	0.64
Hallucinations	194	83.6	135	84.9	329	84.1	0.73
Subjective thought disorder	159	68.5	94	59.1	253	64.7	0.06
Depressed mood	212	91.4	130	81.8	342	87.5	0.005
Anhedonia	203	87.5	115	72.3	318	81.3	<0.001
Suicidal ideation	171	73.7	105	66.0	276	70.6	0.10
Poor concentration	179	77.2	107	67.3	286	73.2	0.031
Irritable Mood	87	37.5	55	34.6	142	36.3	0.56
Elevated Mood	110	47.4	57	35.9	167	42.7	0.023
Lack of insight	47	20.3	49	30.8	96	24.6	0.017
**Males (*n*, %)**						
Delusions	105	92.1	105	92.1	210	92.1	1.00
Hallucinations	95	83.3	99	86.8	194	85.1	0.46
Subjective thought disorder	83	72.8	69	60.5	152	66.7	0.049
Depressed mood	98	86.0	91	79.8	189	82.9	0.22
Anhedonia	95	83.3	76	66.7	171	75.0	0.004
Suicidal ideation	84	73.7	76	66.7	160	70.2	0.25
Poor concentration	77	67.5	73	64.0	150	65.8	0.58
Irritable mood	35	30.7	36	31.6	71	31.1	0.89
Elevated mood	44	38.6	40	35.1	84	36.8	0.58
Lack of insight	26	22.8	38	33.3	64	28.1	0.08
**Females (*n*, %)**						
Delusions	108	91.5	43	95.6	151	92.6	0.38
Hallucinations	99	83.9	36	80.0	135	82.8	0.56
Subjective thought disorder	76	64.4	25	55.6	101	62.0	0.30
Depressed mood	114	96.6	39	86.7	153	93.9	0.018
Anhedonia	108	91.5	39	86.7	147	90.2	0.35
Suicidal ideation	87	73.7	29	64.4	116	71.2	0.24
Poor concentration	102	86.4	34	75.6	136	83.4	0.10
Irritable mood	52	44.1	19	42.2	71	43.6	0.83
Elevated mood	66	55.9	17	37.8	83	50.9	0.038
Lack of insight	21	17.8	11	24.4	32	19.6	0.34

### Physical and Psychological Health Profile

**Table 4** displays the results of the final multivariate logistic regression model for men and women, adjusted for age at interview and socioeconomic status. Most of the physical and psychological health variables that had been significant in the univariate analyses were no longer so in the multivariate models for either gender. However, males who had experienced CTA were more likely to report experiencing a lifetime history of cardiovascular/stroke health issues, migraines and anhedonia. Conversely, females who had experienced CTA were significantly more likely to be in a married/*de facto* relationship and a lifetime history of elevated mood. The physical health measures were not significantly associated with CTA.

**Table 4:** Relationship between CTA and demographics, psychological and physical health profiles in people with psychosis, stratified by sex.

	Males			Females		
	*OR*	95% CI	*p*	*OR*	95% CI	*p*
Age at interview	0.98	0.9–1.0	0.36	1.04	0.9–1.1	0.29
Marital status						
Single, never married	Reference group			Reference group		
Married/*de facto*	–			4.89	1.2–19.2	0.02
Currently separated, divorced, widowed	–			2.05	0.6–7.5	0.28
Lifetime history of smoking	0.41	0.1–2.6	0.34	1.24	0.6–27.3	0.89
Lifetime history of heavy alcohol use	1.43	0.7–3.0	0.34	1.39	0.4–4.4	0.57
Lifetime history of cannabis use	0.93	0.9–1.0	0.07	1.07	0.9–1.2	0.18
Psychological symptoms						
Anhedonia	2.70	1.2–5.9	0.012	–		
Elevated Mood	–			4.11	1.38–12.2	0.01
Physical symptoms						
Cardiovascular/Stroke	4.18	1.5–11.8	0.007	–		
Migraines	3.08	1.2–7.1	0.008	–		

## Discussion

Considerable research has been conducted into the effect of CTA on health, psychological, and social functioning in female populations. Associations between CTA and physical illnesses, including cardiac, respiratory, and gastrointestinal diseases, has also been reported in general population samples (Goodwin and Stein, 2004). However, CTA in male cohorts has received less consideration, primarily due to its seemingly lower prevalence rates and less overt symptomatic presentation(s). The high rate of the self-reporting of physical illness in male psychosis populations such as ours may be evidence of a CTA history. Most significantly, it may characterize a psychological disorder, one that represents a manifestation of an undisclosed CTA experience(s). Self-reported health risks can potentially compound the already high health, psychological, and social burden associated with psychotic illness for this population. These represent deficits that can exacerbate the psychological burden of the experience(s) of CTA. The SHIP cohort presented with a myriad of physical, psychological, and social challenges that did not translate into multivariate explanatory models. Given that the entire sample was in poor physical and psychological health and were socio-economically marginalized, this should not surprise. Overall, prevalence of CTA was high, with over 60% of participants reporting physical, sexual and/or emotional abuse, and neglect. This is consistent with the association between CTA and adult psychotic disorders (Morgan and Fisher, 2007; Matheson et al., 2013). Participants with a CTA history left school earlier and experienced higher rates of employment. Conversely, unlike general population samples where CTA has been associated with higher rates of substance use (Dube et al., 2003), the CTA cohort in this sample did not replicate this research. Furthermore, the CTA cohort was more likely to be married and have children, suggesting a better capacity to form intimate relationships. Additionally, the self-reported poorer physical health, which was more prominent in men, may reflect somatic expression of emotional distress, or poorer self-care with greater vulnerability to physical illness.

Our study accessed a large representative sample of people with psychotic illness living in a disadvantaged region of urban Adelaide, South Australia. The finding that CTA may influence and potentially compromise health functioning among adults with psychosis is intriguing. Despite this, there has been very little research into the assessment and treatment of the effects of CTA in adults with psychosis. Thus, there are unanswered questions about the role of CTA in the symptoms and outcomes of men with psychosis and this awaits future research. Further research is also needed to determine the extent to which the relationship between CTA and health and psychosocial functioning is moderated by other intervening variables, such as cognitive function, substance abuse, and illness symptomology, in psychosis populations. A significant portion of non-psychosis trauma in the literature links CTA to deficits in areas such as psychological and social functioning (Mulvihill, 2005) as well as health functioning (Norman et al., 2006). Nonetheless, our sample unexpectedly did not replicate this association, either at a univariate or multivariate level of analysis. Our findings suggest that interventions are urgently needed. These challenges highlight the importance of an integrated approach to mental health and community based service provision. Such an approach may help to ensure that people with psychosis and the experience(s) of CTA have their health and social needs met in ways that can enhance their illness recovery. Answers to these questions will help in better understanding the processes surrounding health and psychosocial functioning in psychosis populations and to develop and deliver more effective clinical treatments. The population-level data collected as part of the 2010 SHIP research provide a solid empirical foundation to guide health and social policy development and the provision of mental health services regarding many aspects of psychosis/CTA populations.

### Limitations

This study has a number of important limitations. Although we were able to explore the temporal relationship between self-reported physical health and clinical outcomes in adulthood, we were unable to determine severity, frequency, duration and perpetrator of the abuse due to the interview design. Therefore, we may have missed important patterns of association. In addition, we acknowledge that longitudinal studies that collect CTA data prospectively are the optimal design for this type of research.

As our data rely on retrospective self-report, the accuracy may be affected by recall bias. However, a number of studies have demonstrated the validity and reliability of retrospective reports of trauma in psychosis populations. These studies indicated that reports of CTA are stable across time, unaffected by current symptoms, and generally correlate well with other sources of information (Arseneault et al., 2011). Additionally, there is evidence that the retrospective assessment of child abuse tends to underestimate rather than over-report real rates, possibly due to poor memory, denial, or embarrassment. Consequently, our data may underestimate the strength of the relationship between CTA and subsequent outcomes.

## Author Contributions

SS was primarily responsible for the conception of the research upon which this paper is based. CG, LZ, and TA assisted in the development of the ideas for the paper and in refining the final manuscript. SS produced the first draft of the manuscript and submitted the final manuscript and completed the literature search and wrote the main body of the manuscript. TA analyzed the data. SSS contributed to acquisition, analysis and interpretation of the national SHIP data; was part of initial discussions on the substance of this paper, and critically contributed to the draft of the revised version. All authors approved the final manuscript.

## Conflict of Interest Statement

The authors declare that the research was conducted in the absence of any commercial or financial relationships that could be construed as a potential conflict of interest.
