# Corrigendum: Gender Differences in the Physical and Psychological Manifestation of Childhood Trauma and/or Adversity in People with Psychosis

**DOI:** 10.3389/fpsyg.2016.01219

**Published:** 2016-08-29

**Authors:** Shaun Sweeney, Tracy Air, Lana Zannettino, Sonal S. Shah, Cherrie Galletly

**Affiliations:** ^1^Discipline of Psychiatry, University of AdelaideAdelaide, SA, Australia; ^2^School of Nursing and Midwifery, Flinders UniversityAdelaide, SA, Australia; ^3^Neuropsychiatric Epidemiology Research Unit, School of Psychiatry and Clinical Neurosciences Laboratory, University of Western AustraliaPerth, WA, Australia

**Keywords:** childhood trauma and/or adversity, psychosis, self-reported health status, gender, outcomes

Due to an oversight by the authors, the co-author Sonal S. Shah was missed. This has been now added in the author list above and the Author Contributions statement has been revised below.

In the section Materials and Methods, sub-section CHILDHOOD TRAUMA AND/OR ADVERSITY, the whole paragraph should be replaced with:

Questions about the occurrence and nature of CTA were included in the SHIP interview. CTA was coded on the basis of responses to the question: “Were there any other very distressing or traumatic events in your childhood (not including parental separation or divorce, or loss of a close relative)?”

Finally, Health (2012) and Bromfield and Holzer (2008) should be removed from the text and from the Reference List.

The authors apologize for these errors. These corrections do not affect the data or the conclusions contained in the manuscript.

The original article has been updated.

## Author contributions

SS was primarily responsible for the conception of the research upon which this paper is based. CG, LZ, and TA assisted in the development of the ideas for the paper and in refining the final manuscript. SS produced the first draft of the manuscript and submitted the final manuscript and completed the literature search and wrote the main body of the manuscript. TA analyzed the data. SSS contributed to acquisition, analysis and interpretation of the national SHIP data; was part of initial discussions on the substance of this paper, and critically contributed to the draft of the revised version. All authors approved the final manuscript.

### Conflict of interest statement

The authors declare that the research was conducted in the absence of any commercial or financial relationships that could be construed as a potential conflict of interest.

